# Sampling Participants’ Experience in Laboratory Experiments: Complementary Challenges for More Complete Data Collection

**DOI:** 10.3389/fpsyg.2016.00674

**Published:** 2016-05-09

**Authors:** Alan McAuliffe, Marek McGann

**Affiliations:** Department of Psychology, Mary Immaculate College, University of LimerickLimerick, Ireland

**Keywords:** averages, qualitative methods, mixed-methods, phenomenology, validity

## Abstract

Speelman and McGann’s (2013) examination of the uncritical way in which the mean is often used in psychological research raises questions both about the average’s reliability and its validity. In the present paper, we argue that interrogating the validity of the mean involves, amongst other things, a better understanding of the person’s experiences, the meaning of their actions, at the time that the behavior of interest is carried out. Recently emerging approaches within Psychology and Cognitive Science have argued strongly that experience should play a more central role in our examination of behavioral data, but the relationship between experience and behavior remains very poorly understood. We outline some of the history of the science on this fraught relationship, as well as arguing that contemporary methods for studying experience fall into one of two categories. “Wide” approaches tend to incorporate naturalistic behavior settings, but sacrifice accuracy and reliability in behavioral measurement. “Narrow” approaches maintain controlled measurement of behavior, but involve too specific a sampling of experience, which obscures crucial temporal characteristics. We therefore argue for a novel, mid-range sampling technique, that extends Hurlburt’s descriptive experience sampling, and adapts it for the controlled setting of the laboratory. This controlled descriptive experience sampling may be an appropriate tool to help calibrate both the mean and the meaning of an experimental situation with one another.

## Introduction: Two Complementary Challenges

It is something of a trite observation amongst psychologists that not everything that matters can be measured. While a truism, any good psychologist also takes this as a challenge. We are aware, sometimes painfully so, of the limitations of our methods, and the complexity of our subject matter. But good science uses a range of techniques that complement one another and allows us to piece together a multiplex but increasingly coherent understanding of the mind and behavior. While some things cannot be measured, they can be observed and analyzed in rigorous and systematic ways that acknowledge and work within the boundaries of valuable data collection.

Our statistics are part of this toolbox of various methods that we use to build an understanding of psychology. Speelman and McGann (2013) reviewed a number of limitations of the mean as a representation of varied measurements, and the kinds of research designs built around their analysis. Their aim in doing so was not to be pessimistic about the possibility of accurate or valid measurement in psychological science, but to prompt a discussion on the ways in which means or averages have been used uncritically and how their use might be improved as part of a wider effort to sharpen research practices in the discipline.

Speelman and McGann (2013) suggest no single means of improving care or practice with regards to the mean. Rather, a critical attitude that keeps theoretical assumptions in sight and reinforces an awareness of the derived nature of the mean (as opposed to it being assumed a measurement of an underlying parameter) is suggested. Mathematical and methodological techniques help refine the reliability of averages, helping to improve our confidence that an average indicates something important and stable about the data that have been collected. But we must also use varied methodological techniques to critically examine the *validity* of those data.

Speelman and McGann (2013) identify a number of assumptions in play in common use of the mean to summarize performance by an individual or group on a given task. The mean is typically used as an estimate of a “true” value being measured, with variability around that mean being a result of noise or other independent variables unrelated to those addressed in the experiment at hand. There are surely many cases where these assumptions hold true, but Speelman and McGann (2013) note that we should also be prepared to test these assumptions as a matter of common good practice.

We should be sensitive to the possibility that variability around the mean may have something important to tell us about the value of that statistic, and we are in need of techniques that allow us to interrogate such variations. Paying attention to variation in task performance could potentially enable us to validate our measurements, reinforce our interpretations, while also giving us a chance to spot new relevant variables, or other forms of confound.

Part of these efforts after validity involves the use of varied data gathering techniques, making a range of observations that might allow new information to come to the fore, and providing insights into patterns of behavior that might otherwise go unnoticed.

Each variable noticed can potentially be isolated, measured, and its contribution to a given set of performances teased apart through experimental or statistical control – in essence refining the mean being measured, distilling out the particular variable of interest from a complex mixture. There are some variables that have proven very difficult to quantify, isolate, and control, despite there being clear evidence that they play a role in how a person reacts to the task, materials, or situation of our laboratory experiments. In particular, the experience of the situation for participants, what the task or actions involved mean for them as they carry out the task, is something that tends to see little systematic analysis in experimental research, but has been increasingly recognized in recent years (Barrett et al., 2010, 2011). In the rest of the current paper we outline some *prima facie* reasons why a participant’s experience of the laboratory and the apparent meaning of the task for them should be taken seriously. We then review some of the reasons, both historical and scientific, why the systematic collection of data concerning participants’ experience remains relatively rare.

We thus outline two challenges that we suggest are somewhat complementary. On the one hand, the use of the mean in empirical studies demands a set of practices that police its validity. On the other, understanding the meaning of a situation requires the collection of remarkably difficult data – experiential reports – that are quintessentially un-averageable. If we are to test and refine the validity of our data, we will need to be able to find some way of examining variation in measured performance that might fit or diverge from variation in observed experiences. We review a number of different techniques for collecting experiential data and argue that, while useful in their current form, could yet be refined to provide us a more effective means of validating and calibrating measurements in laboratory behavioral experiments. While mixed methods approaches are becoming increasingly prevalent (Tashakkori and Teddlie, 2010), and have been deployed in a wide range of settings (REFS), we suggest that there remains a need for a new form of research method that more closely allies standard laboratory experiments with the collection of reports of participants’ specific experiences of those experiments.

## Validity, Experience, and Experimental Control

Assessing the validity of our measures is made difficult by the fact that it cannot be achieved via a single method. Though we might have a perfectly reliable measure, certainty regarding what it is that we are actually measuring comes not from the consistency of its numbers, but from our understanding of the tool and the ways in which it is used. The understanding that is vital to validity comes from approaching the same phenomenon from other angles, using other methods. No measurement is pure and no experiment perfect, but over time and through the convergence of multiple points of view we gradually develop a picture of our subject matter in increasingly fine resolution. Where validity of the mean, in particular, is concerned, we will need several complementary studies of a behavioral phenomenon that make it clear it is reliable, and insofar as the meaning or experience of the situation is one of the things that cause it to vary, that we sample those as appropriate.

Decades of research in Psychology have taught us that in the experiments where we make our measurements, meaning matters a great deal. Meaning has been on the agenda in some form or another since the “New Look” studies of Bruner and colleagues, which played a substantial role in the rise of cognitive psychology. Bruner and Goodman (1947) reported that coins were perceived or remembered as having different sizes depending on the economic status of the person doing the perceiving, while Bruner and Postman (1949) showed error and expectancy effects due to prior experience and understanding of decks of playing cards. Bruner (1990) has since distanced himself from the computationalist understanding of the mind that developed in part from this line of work on perception, but maintains that understanding the role of meaning in psychology is vital if we are to advance the science, advancing a theory of meaning as culturally enacted but still constitutive of cognitive activity.

The classic work of Treisman (1960), still cited in introductory texts to cognitive psychology, illustrated how people’s attention often moves fluidly with the meaning of the stimuli they are being exposed to, rather than the particular sensory channel on which they were supposed to be focusing. While such research as the New Look and experiments on attention made it clear that the meaning of the stimuli matter for so-called “lower level” aspects of cognition, decades of research were triggered when Wason (1971) showed that it affects reasoning too. People reason to different inferences depending on whether the material they were working with were meaningful to them – whether the materials fit a person’s general experience of the world – or whether they were abstract and contrived.

Perhaps more pointedly, research on participants’ experience of psychological research itself highlights the potency of a situation’s apparent meaning for people’s behavior. Since Orne’s (1962) exploration of demand characteristics, we have been sensitive to the fact that participants who interpret the experiment as testing a particular hypothesis tend to skew their behavior (either deliberately or unconsciously) to support or undermine the perceived hypothesis. Orne (1973) argued that people respond to the “total experimental situation” and that a range of steps should be taken to cope with the rather holistic nature of the setting influencing people. Orne’s work itself developed within a context of increasing disciplinary recognition that the stimulus materials were only part of the picture in understanding behavior in psychology experiments.

Rosenberg (1969) reported three conditions of a study in which participants were asked how much they liked or disliked various pictured persons. Both groups were informed that past research indicated that liking–disliking reactions to strangers correlated with maturity. One group were told that psychologically mature and healthy individuals show greater liking for strangers than immature people and were given fabricated journal article citations. The other experimental group were told the opposite – that research indicated that immaturity was associated with greater liking of strangers, with fabricated journal articles cited. Both groups, however, were informed that they were not going to take part in a study of liking–disliking images of strangers, but rate pictures of strangers to create a standardized list of photographs. Participants believed that these photographs were then going to be used in a liking–disliking task in future research. It isn’t surprising that there were significant differences between the groups, but the obvious manipulation here is not the full story. Rosenberg’s work is a clear illustration of evaluation apprehension, which can be made to affect experimental responding. However, Rosenberg also included a control group with no information about maturity and liking. The results indicated that male participants in this neutral context condition rated male pictures much lower than both experimental groups. They even rated the images substantially lower than the group that were informed that lower ratings was associated with maturity.

Expectancy, social desirability, and demand effects within psychological research are all indications that what participants are doing is not naively fixed by the explicit instructions presented to them, but richly enmeshed with the meaning of the context as a whole. The average response to a given task or stimulus is a product not of a single fix instruction set, but a varied participant-lab situation.

More subtly, work by Gallagher and Marcel (1999) with patients with dyspraxia indicates how their performance on a given task varies substantially with its meaningfulness. Very similar bodily movements that are difficult or impossible for a patient in clinical assessment might be performed relatively smoothly and effectively in situations where the context is more meaningful for them. Lifting a cylindrical object from a table might be a challenge, but taking a drink of water from a tumbler straightforward. Touching their nose on demand can be difficult, but pushing their glasses back into position is done without pause for thought.

More recently we have seen a renewed surge in interest in context, and how it is defined not just by the stipulations of the experimenter but by the total situation involving the thoughts, feelings and behaviors of a particular person, at a particular time (Barrett et al., 2010, 2011; Schwarz, 2010). The experience of the participant and the meaning of the situation for them is once again being acknowledged and given a central role in how we consider their behavior. If we are to adequately understand what a person does, so the understanding goes, we cannot just examine the “input”, the stimuli used, the wording of instructions, or the logical details of the task in which the person was engaged. The validity of our measures is derived from the whole situation and should be examined within the context of that whole situation – including their own experience of it. Though there is no claim that this is *all* that matters, this is one facet of the complexity of a laboratory situation affecting the value and variability of measurements made in that situation, and which should be included as a consideration when policing the validity of those measurements across replications.

Several related threads of theoretical and empirical work share this concern with experience. They tend to vary, however, in terms of their descriptions of the relationships between experience and behavior (Thompson, 2007; Di Paolo, 2009; Shapiro, 2010; Wilson and Golonka, 2013) though most commonly the specifics of that relationship remain ill-defined.

There are thus long threads of research through the history of experimental psychology, including many that have become increasingly influential in recent years, that make a strong case for including some account of the participants’ experience of the experiment in our analyses and interpretation of the data (or at least some aspects of the data). Swinging against this trend, however, is one with an even longer history within the discipline pointing to the weaknesses and unreliability of people’s description of their own thoughts and behavior.

### Good Reasons to Distrust Experiential Reports

While it is clear that people’s experience matters to their behavior, more than a century of research has shown us that it is difficult to understand just *how* it matters. Scientific psychology had the examination of consciousness at its core during the period when all of its major institutions were founded. However, several decades of the analysis of experience ground to a halt in the face of difficulties with introspection. The difficulties of shared analysis, the challenges of independent testing, and the existence of unfalsifiable claims, all made consciousness a problematic notion for a burgeoning science (Watson, 1913; Fancher, 1996; Richards, 2002).

Experience was marginalized by most forms of behavioristic psychology that dominated research through the middle half of the twentieth century. When interest arose again in latter decades, much of the research showed that what effects the meaning of a situation might have for participants’ behavior, can occur without them being consciously aware of it. As such, people are poor describers of their own behavior, or the reasons for it. Perhaps most famously, Nisbett and Wilson’s (1977) review supporting the idea that people have little to no insight into the causes and influences on their own behavior drove home just how poor a source of data individual’s self-report is when we are interested in understanding their actions. Not only does it seem that we do not accurately experience the causes of our actions, but we are happy to invent reasons or explanations that bear little relation to what those real influences are.

Johansson et al.’s (2005) instant classic work on “choice blindness” more recently illustrated just how quickly we can produce such confabulations. Participants, when asked to choose the more attractive between two photographs, and then asked to explain their decision after being handed the *wrong* photo still offered reasons, some mentioning unique aspects of the new (unchosen) picture. Later work showed these confabulated justifications for events to be insensitive to what actually happened (Johansson et al., 2006).

Relatedly, Marcel’s (1993) work on multiple modes of response indicates that we can simultaneously be conscious of a stimulus in one response modality but not in another. That is, if asked to speak a response or press a button, the same stimulus might be simultaneously in a person’s experience and not. Experience, whatever it might be, cannot be understood as a single, simple stream of thought tightly bound to our behavior (Dennett, 1991).

Work in the neuroscience of vision seems to compound this distinction between experience and action through the identification of two apparently quite separate streams of visual processing in the brain (Milner and Goodale, 1995; Goodale and Milner, 2005). One, the dorsal stream, seems specialized for the coordination of visuo-motor action, enabling a person to engage effectively with objects through visual cues. The other, ventral stream, appears to process the visual awareness of objects, dealing with object recognition and naming. Various forms of so-called “blindsight” illustrate the dissociation between these two streams, where a person’s experience can partially or dramatically disrupted while their actions remain effective (Milner and Goodale, 1995).

The consistent trend throughout research on consciousness and behavior is that the linkage between these two aspects of psychology is not straightforward. Understanding that relationship will not come from any casual introspection or direct insight from people reporting what they think. In the existing research the tendency is to explore people’s awareness of their own actions, the reasons for those actions, or in the case of the likes of Marcel’s work, their responses to minimally relevant stimuli – that is images or sounds that only matter to the participant within the constraints of the research task. To that extent the research has tended to focus either on a person’s already conceptualized, considered experience – their *metacognitive* awareness of their thought and actions – or on tasks that are stripped of meaningful context for people and therefore do not fit easily within their normal range of behavior or their normal experiences.

The recent rise in interest concerning context, experience and meaning noted above (see e.g., Varela et al., 1991; Lutz, 2007; Barrett et al., 2010; Mesquita, 2010; Schwarz, 2010; Froese et al., 2011a,b) has criticized such pre-interpreted data. While we must clearly be wary of the claims about their experience and their behavior that we elicit from our participants, there might still be important information we should collect from them about the experience itself. These recent trends lean toward including the analysis of some form of “raw” experience in the interpretation of behavioral data, and perhaps the interrogation of variability within those data. The existing research makes it clear that there is a strong relationship between the participant’s experience, what the situation means to them, and their behavior. It is equally clear that this relationship, however, strong, is complicated. There is no tight coupling between how a person experiences a situation or stimulus, and the fine-grained details of their behaviors in response.

That the existing research leaves us in such a state of confusion suggests that the manner in which we have been collecting data concerning experience is limited, and that other methods are required. We must be careful and nuanced in our gathering and interpreting of experiential reports. While people may provide poor explanations for their actions, their reports of just what they experienced may nevertheless hold valuable information for psychological researchers. Over the past two decades a number of different research methods have developed that may improve matters. We argue that while these methods certainly advance the science of the relationship between experience and action, and can therefore help explore some of the issues regarding variability in behavior on the basis of the meaning of the laboratory situation for the participant, there remains room for refinement.

## Newer Techniques for the Study of Experience: Wide and Narrow Approaches

Different approaches to studying experience come with different commitments to levels of analysis, timescales of measurement, and quality of information regarding the person’s activity at the time of the experience being examined. Some methods, which we will here term ‘wide’ approaches to experience, gather reports or observations in a manner that involves less structure or deliberation with regards to the activity in which the person is engaged at the time, but tends to maximize the range of possible responses and is often captured in ecologically relevant activities.

Examples of such wide approaches are most standard qualitative research methods in psychology, such as interviewing or focus groups (Banister, 2011), the bottom-up explorations of interpretative phenomenological analysis (Reid et al., 2005; Palmer et al., 2010), and descriptive experience sampling (DES; Hurlburt and Heavey, 2002; Hurlburt et al., 2002; Hurlburt and Akhter, 2006), with its randomized triggering of introspective episodes.

Wide approaches gather less constrained information, and in doing so enable a broader exploration of possible research questions. While it is possible to explore the relationship between experience and actions with these methods, this tends to produce a high level, low-resolution picture. These kinds of analyses are useful in pointing us in the direction of more specific research questions, and identifying broader patterns that are difficult if not impossible to see using more narrowly focused methods.

Interviewing and focus groups, for instance, allow us to explore people’s concepts of what they are doing, or how they understand the situation in which they find themselves (Banister, 2011). When participants’ understanding is our key point of interest, this is valid. However, where our interest is in understanding the specifics of the relationship between actions and behavior, things break down, as the classic work on this issue in experimental studies has shown.

Intepretative phenomenological analysis (IPA) is modest in its aims in that it eschews claims to produce facts or unbiased data, but notes that most people are not naive in their experiences – they are experts, or at least familiar with the kinds of situations in which they typically find themselves (Reid et al., 2005). In partnership with a researcher people can reflect upon and interpret their experience using all of the richness of history and context that they bring to the situation, enabling the exploration of certain kinds of relationships unavailable to many more mainstream research techniques. The data typically collected for IPA are interview transcripts, and as such depend on the participants’ recollection for the event or events being examined. Where the particular coupling of experience and behavior is of interest, there are quite strong limits on what kind of insights this form of analysis will enable.

Descriptive experience sampling aims to access “pristine” (Hurlburt and Akhter, 2006) experience, relying less on retrospective accounts of an experience, more on notes and recorded comments made in the moments immediately following an instant of experience, prompted by a beeper device or similar trigger. The pristine nature of the experience – that it is within the flow of the person’s natural activity, sampled without much warning by a randomly occurring trigger – is at the heart of the method’s intended use. Random sampling, and the uncontrolled character of the environment mean that the possibility of associating experiences with particular behaviors is once again limited (though not entirely ruled out, see Hurlburt et al., 2002).

Wide approaches to the study of experience are open to the flow of experience and behavior within naturally occurring activity. In approaches that are both qualitative and mixed methods, these techniques have been applied in domains such as Nursing (e.g., Traylen, unpublished MPhil dissertation), Education (e.g., Onwuegbuzie et al., 2007; Palak and Walls, 2009), Anthropology (e.g., Killick, 1998), as well as Psychology (e.g., Hurlburt and Akhter, 2006). They offer useful insights into the relationship between experience and behavior, and can be used to help structure sequential mixed methods research projects where concepts and experiences are sampled in ecologically rich settings and then variables identified for closer inspection in laboratory experiments. For the more fine-grained examination of specific variability of behavior in those experiments, however, these approaches tend to be too broad, examining timescales that are too long to adequately sample experience at the grain of analysis that the behavior is being measured.

“Narrow” approaches, on the other hand, focus more particularly at the level of momentary experience and momentary behaviors. In a sense, the entire domain of psychophysics exists at this level of analysis, a very longstanding and finely tuned examination of the relationship between physical stimuli and a person’s experience of them. A somewhat related but distinct precedent in the methodological literature is that of systematic observation (Hintze et al., 2002; Podsakoff et al., 2003). Systematic observation, with a long history in various disciplines, clearly specifies the behaviors of interest in advance and observes them (and only them) in naturalistic settings. It therefore constitutes as more focused form of observation than the “wide” approaches outlined above. The technique tends not to involve the sampling of participants’ experience or awareness of their surroundings at the moment of interest, however, and the measurements of behavior while specific, are typically more coarsely grained than would be common in controlled experiments (though this may change as technology advances).

In the present paper, our interest is specifically with the experience-behavior relationship, and how variability in experience might be used to better understand variability in measured behaviors. For that purpose we find two candidate approaches in recently developed methods for fine-grained experiential data collection: neurophenomenology (Varela, 1999; Lutz and Thompson, 2003; Thompson et al., 2005) and the elicitation interview (Petitmengin, 2006; Petitmengin et al., 2013).

Both neurophenomenology and the elicitation interview involve quite substantial control over the environment in which that data are collected. In the case of neurophenomenology the research is conducted in a neuroscience laboratory, usually with EEG recording, and involves the careful training of participants in phenomenological introspective techniques (that is, introspection that attempts to avoid conceptualisation of the experience, but to review and report it in as close to an atheoretical fashion as possible). Neurophenomenology is thus an example of a mixed methods approach (Tashakkori and Teddlie, 2010; Creswell and Plano Clark, 2011), seeking calibration of quantitative measures with qualitative reports. The elicitation interview is similarly conducted in a controlled setting, but in this case the participant is not trained to introspect but interviewed by a specialist in a manner intended to evoke the experience of a particular moment, as opposed to some particular *post hoc* understanding of that moment.

Being lab-based, both neurophenomenology and the elicitation interview offer the possibility of linking experience with reliably, and finely, measured behaviors. They provide the possibility of a high resolution examination of the relationship between experience and action. They are not, of course, without their drawbacks.

Neurophenomenology requires training of participants in the particular introspective techniques associated, and in doing so alters the very experience we are studying. Lutz and Thompson (2003) argue that this is not a deep problem, though do not offer a full explanation as to why. While it is quite possibly true that coming to an understanding of experience will necessarily change it, we would argue that methods should still be explored that might possibly provide us with naive or unreflective experiential reports. We do not argue against neurophenomenology, but simply note that there may yet be useful experiential data to collect from participants whose reports are not pre-disciplined by the training they have received. Neurophenomenology is one tool available to us, we note that others are yet needed.

The elicitation interview purports to provide just such naive data, and in this we see real promise, but two facets of the technique imply limits that might still leave us with an important methodological blindspot.

The collaboratively constructed nature of the interview process is one point of consideration, keenly aware as we already are about the ease with which apparently confabulated responses about experiential reports are produced. While proponents of the elicitation interview approach argue strongly that a properly skilled interviewer neither foists particular descriptions nor prompts invented reports from their interviewees (Petitmengin, 2006), we must yet proceed with care. This means that the approach, while both demanding of extraordinary discipline on the part of the interviewer and substantial time for its conduct (often between half an hour to an hour per interview), must still be used with caution. Such pragmatic considerations must not stop us from doing good science, but they do, nevertheless, motivate us to be fully cognisant of the range of choices we have available.

More concerning for our current purposes is the standard focus of the elicitation interview: the re-evocation of a particular moment of experience, an instant, as it were, during which a decision was made, or a response to a question as it popped into the interviewee’s mind. The techniques of the interview bring the participant back to that moment, as though it were as real and rich as their immediate environment. With the previous experience thus being relived, it can be interrogated in fine detail. In doing so, however, the temporal relationship between event and subsequent discussion is broken. In Petitmengin et al.’s (2013) recent study on the Johansson et al.’s (2005, 2006) choice blindness task, for instance, some participants completed the photo choice and explanation at the normal pace, with reports on the decision occurring between 5 s and 1 min after the choice. The elicitation interview involved a period of between 30 and 45 min post-decision before re-presentation of the photo and evoking of explanation. It is very likely that the collection of systematic experiential reports of any kind is going to involve the interruption of the flow of behavior within a task in some form. We would argue, however, that more modest interruptions should be more attractive, and where possible the temporal dimensions of the task should be carefully balanced across participant groups. What is more important, however, is the possibility of multiple sampling points throughout the course of a task. Where highly focused techniques such as the elicitation interview provide fine-grained examination of a single moment, there is not only a possibility but some suggestive evidence of multiple strands of experience, and multiple rhythms of attention or endogenous sensitivity to different aspects of the environment operating over different timescales (Varela et al., 1981; Donald, 2001; Busch et al., 2009). That is, our experience is not just a string of beads, but has multiple tempos and currents to it that will need multiple sampling to observe, a form of repeated probing that the likes of the elicitation interview makes unfeasible.

We therefore argue that there is room between the wide and narrow forms of investigation of experience for a set of intermediate methods. This intermediate range is more anchored in recorded events and actions than wide approaches. Such an approach will enable it to be used within controlled environments, and thus offers promise in collecting data relevant to the interrogation of variable behavior in controlled settings. The approach would also, though, be less finely coupled to particular stimuli or instants of experience than the more narrow approaches. The meaningfulness of actions is to be sampled at this intermediate range, where we might find patterns of behavior rather than individual events, and themes of experience rather than fine-grained particulars. Instead of the fast, very short durations of most neural events as measured and used in neurophenomenology, we might explore the slower, 10s of seconds or minutes of duration in common behavior settings. Given the history of research on experience-behavior links, we might expect relationships between sampled experience and behavior to need this kind of re-sampling, so that variability in behavior can be calibrated against variability in experience, rather than trying to capture something fixed in either one.

## Suggesting an Intermediate Level of Analysis

While dependencies of behavior on a host of contextual factors is violated in laboratory experiments, this is a compromise adopted for the purposes of maximizing communicability (through standardized meanings to terms and procedures) as well as replicability [an issue of some current concern amongst researchers (Koole and Lakens, 2012; Nosek et al., 2012; Open Science Collaboration, 2012, 2015; Ritchie et al., 2012; Roediger, 2012)].

Long running debates over the value of lab vs. field research are essentially the professional policing of this compromise, an exercise in maintaining perspective on the complementary values of different forms of data collection, and an effort at continually refining and improving our methods. The collection of reports of the experiences of participants is no exception to this issue, with wider approaches serving richer understandings of context, while the more narrowly focused techniques offer higher resolution accounts of more finely circumscribed phenomena. Wide approaches explore the general attitudes and experiences of a person at a conceptual level that fits the person’s understanding of their situation and actions, but that makes specific reference to particular experiences and behaviors challenging. Narrow approaches, on the other hand, may in fact be swamping the signal on the relationship between experience and behavior with the noise of momentary stream of consciousness, much of which is irrelevant to the niceties of bodily action (Aglioti et al., 1995; Milner and Goodale, 1995). If the meaning of the situation (as suggested by the likes of Barrett et al., 2010), rather than strings of isolated stimuli, are part of what matter to the structuring of behavior, and the variability of measurements around a mean for a given behavioral variable, then at least some of the varied methods we use should be calibrated at that appropriate scale.

Without knowing what experiential data most matter for best understanding behavior, the wise course of action is to sample widely and often, but within a setting where the behavior is sufficiently reliable to keep subtle relationships stable (or as stable as they can be). We suggest a form of controlled descriptive experience sampling (a “C-DES”), where introspective moments are triggered as with standard DES – without prior warning to the participant, via a beep or flash, perhaps. The participants might understand these triggers to be random, but they need not be in actuality. Descriptions can be kept brief, to potentiate multiple such sampling during a single task or event as appropriate. Further, the purely verbal descriptions of standard DES might also be augmented with simple video recording of non-verbal behaviors such as blinks, eye-movements, or other possibly subtle, aspects of the participant’s behavior, offering a richer interpretative context for the content of reports (Olivares et al., 2015).

To offer an illustration, the Iowa Gambling Task (Bechara et al., 1994) is a frequently used laboratory activity conducted to evaluate participants’ sensitivity to certain kinds of consequences, or to investigate trait characteristics such as impulsivity or executive control. The task is sometimes augmented with questions to the participant about their knowledge of its various components, to see how this changes over the course of the activity. Just what the relationship between participants’ knowledge and their behavior is over the course of the task is somewhat problematic, but C-DES would eschew a need for the *participant* to understand the task at all, or report knowledge of it. Rather, by sampling what they were aware of either at key moments, or at regular intervals over the course of the task, researchers might be able to explore this relationship without relying on participant insight.

While this runs counter to the standard use of DES, for which naturalistic activity is vital, many of the strengths of the approach are maintained (no pre-specification or priming of behavior or moment to be introspected upon, naturalistic description of experience by participants). These strengths might thus be deployed in the service of understanding people’s experiences of the laboratory during the laboratory task, and provide one of several perspectives from which we build up a richer understanding of what people are doing, and how they are experiencing the doing of it.

We will not know without conducting the research what kinds of experience will be relevant. History indicates clearly that introspective explanations of behavior are not the data we are looking for, but a plethora of other options are available, across numerous scales of time. Sensory experiences, physiological rhythms and responses, emotions, moods, culturally relevant routines – these things, and more show up in people’s descriptions of their experience. While long-practiced habits might primarily shape behavior at the level of momentary particulars, experience may instead be coupled with action at the level of “molar behavior” (Barker, 1968).

This is to say that experience may not be a flow of individual moments in continuous accumulation, but a general awareness of a situation within which various relationships become distinguished – an event does not simply happen at some psychological “now”, but early or late within a general expectation or understanding of the setting. Longstanding (but little known) work indicates that people are very sensitive to the standing patterns of behavior or expected routine present within a given physical or social setting (Barker, 1968; Schoggen, 1989; Heft, 2001, 2003, 2007; see also Heft et al., 2014, for a recent examination of people’s ability to recognize settings with very limited information). The work of Mesquita (2010) and Barrett et al. (2011, 2014), have shown a similarly situational character to people’s emotional reactions.

Within a more controlled form of DES the probing of conscious awareness can remain open and largely unstructured. Participants are free to describe their experience in familiar and comfortable terms, which can be explicated in conversation with the experimenter either immediately, or at a later time after the experimental task itself is completed. For the main, the standard DES principles outlined by Hurlburt et al. (2002) apply. The time between experience reporting and exploration in collaboration with the researcher is very short. Moments of experience are clearly defined (by the use of a tone or other trigger). Various practices of the interview are used to ensure that careful distinctions are made between the experience itself and any attempt to explain that experience.

In addition, however, given that the initial probings of experience can be kept brief (or varied in length depending on research goals), the possibility of multiple samplings over the course of a single experimental session is maintained. The intervals between samplings can be used as a means of exploring the temporal aspects of experience, its rhythms, and periodic variations.

## Using the Un-Mean-Able to Calibration the Mean (and Vice-Versa)

Focusing closely on averages as summaries of collections of data is a practice that depends on a host of background theoretical assumptions. Speelman and McGann (2013) raised concerns (oft-noted in statistics courses, but rarely applied in practice) that these assumptions are commonly unquestioned, and frequently ill-considered. While there are some reporting and analysis practices that might help contextualize the mean in mathematical or statistical terms, and we support calls to move toward standardizing such practices (such as Doherty et al., 2013), it is equally important to query the psychological, and not just statistical, context to the data being collected.

In this paper we have argued that there are good reasons for paying more attention than we typically do to the experience of the participant within rigorous laboratory experiments. There is clearly a relationship between participants’ experiences of a given situation and their behavior within that situation, but the relationship is not a simple one. The validity of our measures, and relatedly our understanding of their variation, must be achieved through the coordination of multiple sources of knowledge about a person and their actions in a given setting. Experiential data, however, challenging they are to work with, have some role to play in that validation and calibration process (Froese et al., 2011a).

What we have termed “wide” approaches to such experiential data collection do not provide us with the behavioral data at the level of detail we need to effect this calibration. Conversely, the approaches we have termed “narrow” we suggest are *too* narrow. Though they enable the collection of specific behavioral data, the pre-focused nature of their experience sampling imposes expectations or prior understandings of the kinds of experience we need to probe, and include assumptions about the momentary nature of those experiences, that are inappropriate for our current levels of understanding (or perhaps more accurately, ignorance), about the behavior-experience relationship, particularly of the varying timescales of different phenomena of consciousness.

We propose that a C-DES is a data collection technique ideal for the kinds of disciplined exploratory research that is needed to adequately observe the experience-behavior relationship. In order to determine to what degree a calculated mean actually matters to what people do, and how to refine the validity of what it measures, we need a level of description and analysis of experiential data that is not commonly in use – one that is exploratory and potentially wide-ranging, but evoked within a controlled, managed situation such as the laboratory experiment. The paired examination of controlled behaviors still offers us a means of understanding and interpreting the descriptions of experiences captured through this process. The validation of the mean and the un-meanable is a two-way relationship, achieved not through a single ideal study, but through a long process of negotiation across multiple studies, using multiple methods.

## Author Contributions

All authors listed, have made substantial, direct and intellectual contribution to the work, and approved it for publication.

## Conflict of Interest Statement

The authors declare that the research was conducted in the absence of any commercial or financial relationships that could be construed as a potential conflict of interest.
